# Secretoglobin Superfamily Protein SCGB3A2 Deficiency Potentiates Ovalbumin-Induced Allergic Pulmonary Inflammation

**DOI:** 10.1155/2014/216465

**Published:** 2014-08-27

**Authors:** Taketomo Kido, Mitsuhiro Yoneda, Yan Cai, Tsutomu Matsubara, Jerrold M. Ward, Shioko Kimura

**Affiliations:** ^1^Laboratory of Metabolism, National Cancer Institute, National Institutes of Health, Building 37, Room 3106, NIH, 9000 Rockville Pike, Bethesda, MD 20892, USA; ^2^Institute of Molecular and Cellular Biosciences, The University of Tokyo, Tokyo 113-0032, Japan; ^3^Department of Anatomy, Graduate School of Medicine, Osaka City University, Osaka 545-8585, Japan; ^4^Global VetPathology, Montgomery Village, MD 20866, USA

## Abstract

Secretoglobin (SCGB) 3A2, a cytokine-like secretory protein of small molecular weight, which may play a role in lung inflammation, is predominantly expressed in airway epithelial cells. In order to understand the physiological role of SCGB3A2, *Scgb3a2^−/−^* mice were generated and characterized. *Scgb3a2^−/−^* mice did not exhibit any overt phenotypes. In ovalbumin- (OVA-) induced airway allergy inflammation model, *Scgb3a2^−/−^* mice in mixed background showed a decreased OVA-induced airway inflammation, while six times C57BL/6NCr backcrossed congenic *Scgb3a2^−/−^* mice showed a slight exacerbation of OVA-induced airway inflammation as compared to wild-type littermates. These results indicate that the loss of SCGB3A2 function was influenced by a modifier gene(s) in mixed genetic background and suggest that SCGB3A2 has anti-inflammatory property. The results further suggest the possible use of recombinant human SCGB3A2 as an anti-inflammatory agent.

## 1. Introduction

Secretoglobin (SCGB) 3A2 belongs to the SCGB gene superfamily of cytokine-like secretory proteins of small molecular weight (~10 kDa) [1–3]. Most SCGB members are found at high concentrations in secretions such as lung, lacrimal gland, salivary gland, prostate, and uterus, yet their biological functions are largely unknown [1]. In lung, SCGB3A2 is predominantly expressed in the epithelial cells of trachea, bronchus, and bronchioles. Its expression is found at the growing tips of bronchi around embryonic (E) day 11.5 of mouse gestation [4], and its level reaches maximum at the end of gestation [5]. SCGB3A2 plays a role in embryonic lung development as demonstrated by* ex vivo* embryonic lung organ culture in the presence of SCGB3A2 and* in vivo* administration of SCGB3A2 to pregnant female mice, followed by examination of preterm pups [4]. In addition, SCGB3A2 exhibits anti-inflammatory and anti-fibrotic activities [6, 7]. SCGB3A2 may also be used as a marker for pulmonary carcinomas [8, 9]. Thus SCGB3A2 appears to possess multiple activities.

The anti-inflammatory function of SCGB3A2 was initially suggested by the following observations: (1)* Scgb3a2* mRNA levels were reduced in the lungs of fungal-induced allergic inflammation model mice, which was almost restored by dexamethasone treatment [6], (2) reduced levels of lung* Scgb3a2* mRNA in the OVA-induced inflammation model mice were inversely correlated with the increased levels of proinflammatory cytokines, IL-5 and IL-9 in bronchoalveolar lavage fluid (BALF) [10, 11], and (3) intranasal instillation of IL-5 or IL-9 to naïve mice reduced* Scgb3a2* gene expression in the lung [10, 11]. Further, when OVA-airway inflammation model mice were intranasally administered recombinant adenovirus expressing SCGB3A2 before OVA challenge, OVA-induced airway inflammation was suppressed with airway overexpression of SCGB3A2 [6]. The latter results unequivocally demonstrated that SCGB3A2 has anti-inflammatory function.

In order to further obtain insights into the physiological functions and anti-inflammatory activity of SCGB3A2,* Scgb3a2* ^−/−^ mice were produced and characterized and subjected to the OVA airway inflammation model [6].* Scgb3a2* ^−/−^ mice in mixed genetic background showed an exacerbation of OVA-induced airway inflammation. In contrast, six times C57BL/6NCr backcrossed congenic* Scgb3a2* ^−/−^ mice exhibited a slightly increased inflammation as compared to their respective wild-type littermates. These studies suggested the importance of genetic background and that SCGB3A2 has protective role in the lung as an anti-inflammatory agent.

## 2. Materials and Methods

### 2.1. Generation of* Scgb3a2* ^−/−^ Mice

**Figure 1:**
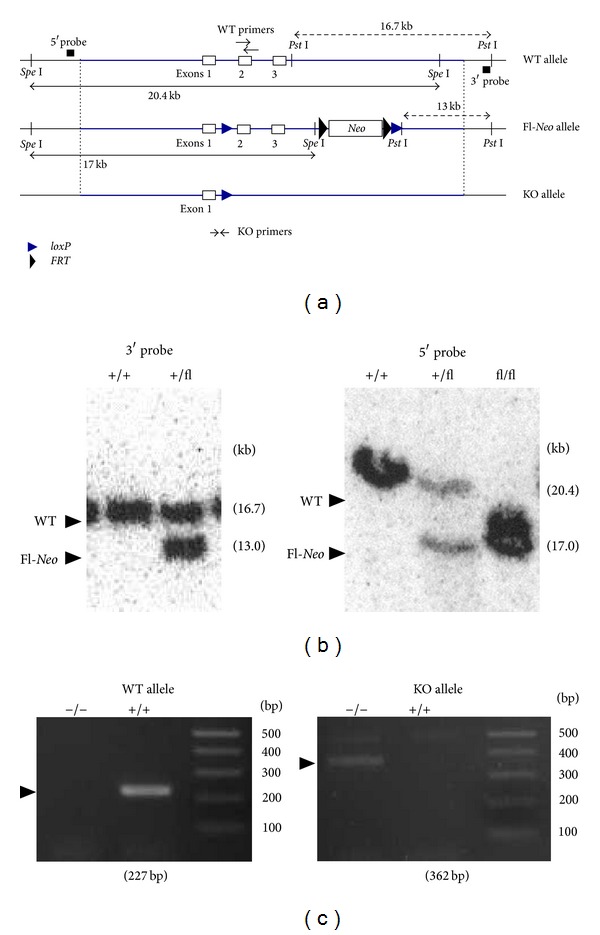
Generation of* Scgb3a2* knockout mice. (a) Schematic of wild-type (top), floxed-*Neo* (middle), and knockout (bottom) alleles after Cre-mediated excision of exons 2 and 3.* Scgb3a2* exons 1, 2, and 3 are indicated by boxes. (b) DNA from wild-type ES cells (+/+) and floxed cell clone (+/fl) were digested with* Pst* I and subjected to Southern blot hybridization using the downstream of the 3rd exon as a 3′ probe (left panel). Tail DNA from F1 progeny of two single floxed*-Scgb3a2* (+/fl) intercrosses was digested with* Spe* I and subjected to Southern blot hybridization using the sequence upstream of the 1st exon as a 5′ probe (right panel). The genotype for the* Scgb3a2 *locus was indicated above each lane. Sizes of the DNA fragments were indicated on the right in the parenthesis. Restriction sites and location of the 3′ and 5′ probes are indicated in (a). (c) Double floxed-*Scgb3a2 *(fl/fl) mice were crossed with EIIA-*Cre* transgenic mice expressing Cre in germ cells. Tail genome from F1 progeny of two heterozygote intercrosses was genotyped by PCR using wild-type (WT) primers and knockout (KO) primers to amplify either the WT (227 bp long, left panel) or the KO allele (362 bp long, right panel). The genotype for the* Scgb3a2 *locus was indicated above each lane. Primer positions are indicated in (a).

Generation of* Scgb3a2* ^−/−^ mice was carried out using homologous recombination of a targeting vector generated by recombineering, followed by embryonic stem (ES) cell injection (Figure 1(a)). During production of* Scgb3a2* ^−/−^ mice,* Scgb3a2-*conditional mice with the* Scgb3a2-*floxed allele were also generated, which were not used in this study; the details will be described elsewhere. Recombineering was performed as described [12]. In brief, 13 kbp DNA fragment containing the mouse* Scgb3a2* gene was subcloned into PL253 vector. The first* loxP* site was introduced in the first intron of the* Scgb3a2 *gene. A Neomycin (*Neo*) cassette flanked by two* FRT* sites and one* loxP* site was inserted downstream of the* Scgb3a2 *gene using PL451 vector that contains this cassette. After these successive recombineering procedures, the final targeting vector was purified and electroporated into C57BL/6J x 129/SvJae hybrid ES cells [13]. G418-resistant ES cells were genotyped by Southern blotting using 5′, 3′, and* Neo* probes. Two correctly targeted ES clones were injected into blastocysts obtained from C57BL/6NCr mice. Southern blotting and PCR analyses confirmed germline transmission in the offspring of* Scgb3a2*-targeted chimeric mice crossed with wild-type C57BL/6NCr mice. The mice were crossed to EIIA-*Cre* transgenic mice (FVB background) to remove* loxP* site to generate a complete knockout allele [14], while *β*-actin-driven* Flp* transgenic mice were used to remove the* Neo* cassette to produce the* Scgb3a2*-floxed allele [15]. The PCR primers for detecting knockout allele were UG1KO-F: 5′-ATC CTC GGG GAA AAG TTC TG-3′ and UG1KO-R: 5′-CTA AAA TCA GGG GCC AGA CA-3′, with a 362 bp product. The primers to detect exon 2 of the* Scgb3a2* gene (for wild-type and floxed alleles) were UG1EX2-F: 5′-ACC GTC TCC CTG TTG TTG AC-3′ and UG1EX2-R: 5′-CAC GTA GCA AAG GCT TCT CC-3′, with a 227 bp product. The mice used in this study were those with mixed genetic background and those backcrossed to C57BL/6NCr for six generations and their respective wild-type littermates. Mice were maintained under standard specific-pathogen-free conditions.

### 2.2. Southern Blot Analysis

Genomic DNA was isolated from tail biopsy and digested with* Spe* I for 5′ probe or* Pst* I for 3′ probe. Digested DNA fragments were run on a 0.4% agarose gel overnight and were transferred to a nylon membrane. ^32^P-Labeled (PerkinElmer, Waltham, MA) probes were prepared with Ready-To-Go DNA Labeling Beads (-dCTP) (GE Healthcare Life Sciences, Piscataway, NJ). Signals were detected using Strom 840 (GE Healthcare Life Sciences).

### 2.3. Western Blotting

Lungs were homogenized and lysed in RIPA Lysis Buffer (Santa Cruz Biotechnology, Santa Cruz, CA) supplemented with complete protease inhibitor cocktail (Roche Applied Science, Indianapolis, IN). Protein samples were separated in SDS-polyacrylamide gels, transferred to Immobilon-P Membranes (EMD Millipore, Billerica, MA) in Tris-Glycine-Methanol Transfer Buffer, and blocked in 5% nonfat dried milk in TBST (50 mM Tris-HCl, pH 7.4, 150 mM NaCl, and 0.1% Tween-20) for 1 hour prior to incubation with primary antibodies at 4°C overnight. The anti-mouse SCGB3A2 antibody (rabbit) was previously described [3]. The anti-GAPDH antibody 6C5 (EMD Millipore) was used as a loading control. Subsequently, blots were washed in TBST and then incubated at room temperature for 1 hour with HRP-linked secondary antibody (Cell Signaling Technology, Boston, MA). Blots were washed in TBST and were visualized with SuperSignal West Dura Extended Duration Substrate for SCGB3A2 and SuperSignal West Pico Chemiluminescent Substrate for GAPDH (Thermo Fisher Scientific, Rockford, IL).

### 2.4. Animal Model

Female mice (6-week-old; 4–8 mice/group) were immunized with weekly intraperitoneal injection of OVA (Sigma-Aldrich, St. Louis, MO; 20 *μ*g in a total volume of 100 *μ*L saline) emulsified with an equal volume of aluminum hydroxide adjuvant (Imject Alum, Thermo Fisher Scientific) on days 0, 7, and 14. OVA (1% w/v diluted in saline) challenge inhalation exposure was administered by PARI LC plus nebulizer and Vios compressor (PARI Respiratory Equipment, Inc., Midlothian, VA) using PIE cage (Braintree Scientific, Inc., Braintree, MA) on days 21, 22, and 23. OVA-injected and saline-challenged mice were used as control. Bronchoalveolar lavage (BAL), followed by collection of lung tissues, was carried out on day 25. Cytospin preparations of BAL fluid were centrifuged onto glass slides through Shandon Cytofunnels (Thermo Fisher Scientific) at 1000 rpm for 5 min. Cell differentiation was performed microscopically over 200 cells stained with Giemsa (Sigma-Aldrich). All experiments were carried out following guidelines for animal use issued by the National Institutes of Health and approved by the National Cancer Institute (NCI) Animal Care and Use Committee.

### 2.5. Histopathology

The lung was inflated with 10% buffered formalin, embedded in paraffin, and cut into 5-*μ*m sections. Sections were deparaffinized and stained with hematoxylin and eosin (H&E). Lung lesions (numbers of eosinophils, bronchiolar mucus, and perivascular and peribronchiolar cuffing with inflammatory cells) were graded histologically in blind fashion as 0, no lesions; 1, minimal lesions; 2, mild; 3, moderate; 4, severe. The histological grade depended on the extent of the lesion in the lung and the severity of the lesion itself. Immunohistochemical staining for SCGB3A2 (antibody dilution 1 : 10000) [3] was carried out on 5-*μ*m paraffin embedded sections by avidin-biotin-peroxidase complex method with VECTASTAIN Elite ABC Kit (Vector Laboratories, Burlingame, CA). The immunoreactivity was visualized by 3, 3′-diaminobenzidine tetrahydrochloride (Dako, Carpinteria, CA) and counterstained with hematoxylin.

### 2.6. Quantitative RT-PCR (qRT-PCR) Analysis

Lung total RNAs were isolated using TRIzol (Life Technologies, Rockville, MD, USA). Oligo(dT)-primed cDNAs were reverse-transcribed from 2.0 *μ*g total RNAs by using SuperScript III Reverse Transcriptase (Life Technologies) according to the supplier's protocol. Real-time RT-PCR analysis was performed using the ABI Prism 7900HT sequence detection system (Life Technologies). Primer sequences used for qRT-PCR are shown in Table 1.* Ppia* (peptidylprolyl isomerase A (cyclophilin A)) was used as an internal control for SYBR Green PCR, which was performed in a single tube in triplicate. The results were expressed as the average of three independent experiments.

### 2.7. ELISA Assay

Mouse IL-4, IL-5, and IL-13 levels were determined by ELISA kits from R&D Systems (Minneapolis, MN, USA) according to the manufacture's protocol. Detection limit was about 2, 7, and 1.5 pg/mL, respectively.

### 2.8. Data Analysis

Data are expressed as means ± SD. Levels of significance for comparison between samples were determined by Student's* t*-test distribution or one-way ANOVA using Graph Pad Prism (La Jolla, CA). *P* values of <0.05 were considered to be statistically significant.

## 3. Results

### 3.1. Generation of* Scgb3a2* ^−/−^ Mice

To understand the role of SCGB3A2 in OVA-induced allergic airway inflammation,* Scgb3a2* ^−/−^ mice were generated in which the 2nd and 3rd exons of the* Scgb3a2* gene were targeted (Figure 1(a)). After homologous recombination in ES cells, a positive clone containing a* loxP*-flanked (floxed-Neo)* Scgb3a2* allele was identified (Figure 1(b)). To produce a knockout allele, these mice were crossed with EIIA-*Cre* transgenic mice that express Cre recombinase at the zygote stage [14]. The deletion of the 2nd and 3rd exons of* Scgb3a2* gene was confirmed by PCR (Figure 1(c)).

### 3.2. Gross Characterization of* Scgb3a2* ^−/−^ Mice

**Figure 2:**
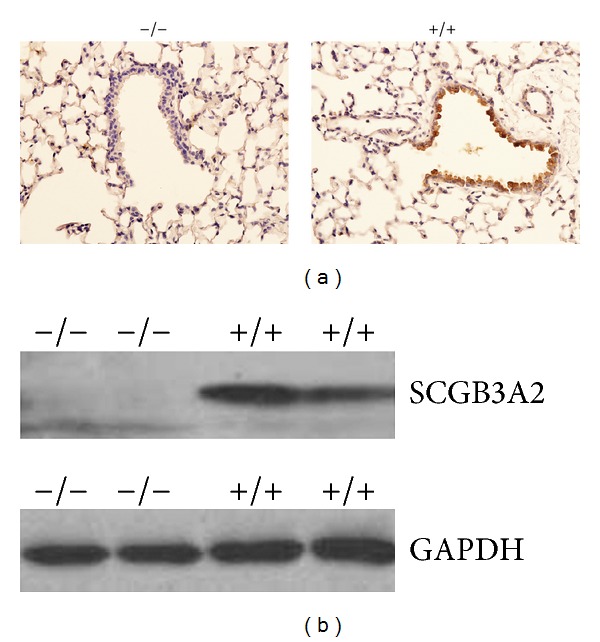
Lack of SCGB3A2 protein expression in *Scgb*3*a*2^−/−(N6)^ mouse lung. (a) Immunohistochemistry for SCGB3A2 of the lung from adult *Scgb*3*a*2^−/−(N6)^(−/−) and wild-type littermates (+/+). In wild-type lung, a robust expression of SCGB3A2 was detected in bronchial epithelial cells shown in brown. Sections were counterstained with hematoxylin. Magnification 400x. (b) Western blot analysis of protein extracts from lung homogenate was performed with the anti-SCGB3A2 antibody. Blotting with anti-GAPDH antibody was used as control for protein loading.

*Sc* *gb*3*a*2^−/−^ mice were healthy and fertile for at least a year, regardless of their genetic backgrounds. They grew at a normal rate and did not exhibit any gross abnormalities or clinical illness. Their lungs when examined at 3 months of age were histologically normal (Figure 2(a) and data not shown). Western blot and immunohistochemical analyses using lung tissues showed that* Scgb3a2* ^−/−^ mice lacked the expression of SCGB3A2 (Figures 2(a) and 2(b)). The knockout mice were backcrossed to C57BL/6NCr six times (*Scgb*3*a*2^−/−(N6)^). They were, together with mixed genetic background mice (*Scgb*3*a*2^−/−(mix)^), used for further experiments with their respective wild-type littermates as control.

### 3.3. OVA-Induced Airway Inflammation Model

**Figure 3:**
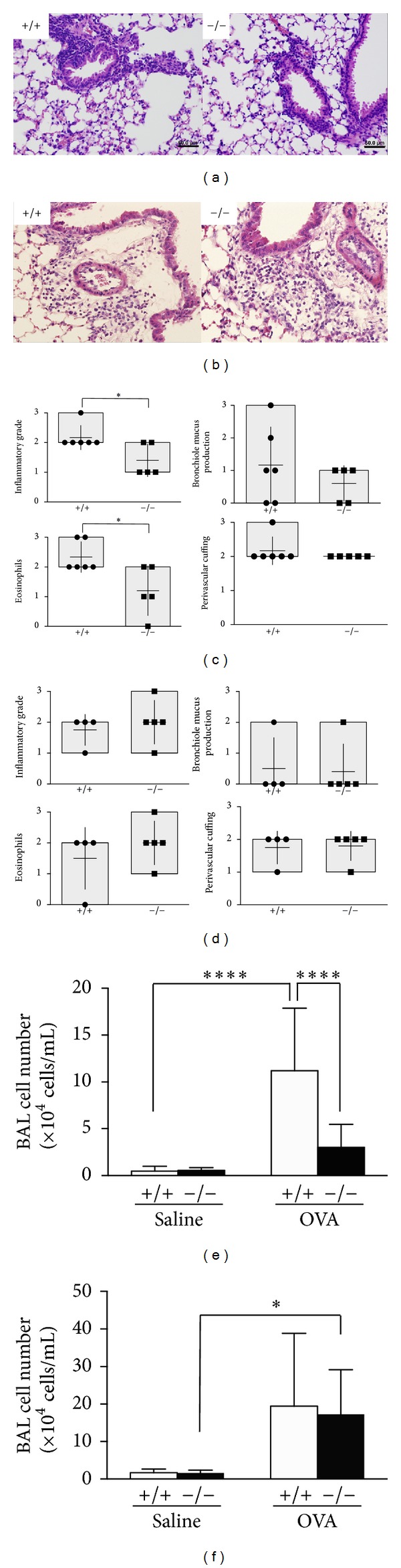
OVA*-*induced airway inflammation. ((a) and (b)) Representative lung H&E stained sections of OVA inhalation challenged *Scgb*3*a*2^−/−(mix)^  (−/−) and *Scgb*3*a*2^+/+(mix)^ (+/+) mice (a), and *Scgb*3*a*2^−/−(N6)^(−/−) and *Scgb*3*a*2^+/+(N6)^ (+/+) mice (b). There is mild peribronchiolar and perivascular cuffing with inflammatory cells. Magnification 400x. ((c) and (d)) Degree of OVA-induced airway inflammation was determined using inflammatory grade, bronchiolar mucus production, infiltrating eosinophils, and perivascular and peribronchiolar cuffing as described in Section 2 for *Scgb*3*a*2^−/−(mix)^(−/−) and *Scgb*3*a*2^+/+(mix)^ (+/+) mice (c), and *Scgb*3*a*2^−/−(N6)^(−/−) and *Scgb*3*a*2^+/+(N6)^ (+/+) mice (d). ((e) and (f)) Analysis of BALF inflammatory cell numbers from *Scgb*3*a*2^−/−(mix)^(−/−) and *Scgb*3*a*2^+/+(mix)^ (+/+) mice with OVA challenge (OVA) as compared with saline challenge (Saline) (e), and *Scgb*3*a*2^−/−(N6)^(−/−) and *Scgb*3*a*2^+/+(N6)^ (+/+) mice with OVA challenge (OVA) as compared with saline challenge (Saline) (f). *P < 0.05 and ****P < 0.0001 by Student's* t*-test for (c) and (d) and one-way ANOVA for (e) and (f).

*Scgb3a2* ^−/−^ mice and their wild-type littermates in both mixed genetic backgrounds and six times C57BL/6NCr backcrossed were subjected to OVA inflammation model. Histologically, the incidence and grading of all lesions were lower with statistical differences in mixed background* Scgb3a2* ^−/−^ (*Scgb*3*a*2^−/−(mix)^) than their wild-type littermates (*Scgb*3*a*2^+/+(mix)^) (Figures 3(a) and 3(c)), while they were at similar levels in both *Scgb*3*a*2^−/−(N6)^ and their wild-type littermates *Scgb*3*a*2^+/+(N6)^ of OVA-challenged mice (Figures 3(b) and 3(d)). The number of inflammatory cells in bronchoalveolar lavage fluid (BALF) was lower in OVA-challenged *Scgb*3*a*2^−/−(mix)^ mice than *Scgb*3*a*2^+/+(mix)^ mice with statistical significance (Figure 3(e)), while there were no obvious differences between *Scgb*3*a*2^−/−(N6)^ and *Scgb*3*a*2^+/+(N6)^ after OVA challenge (Figure 3(f)).

**Figure 4:**
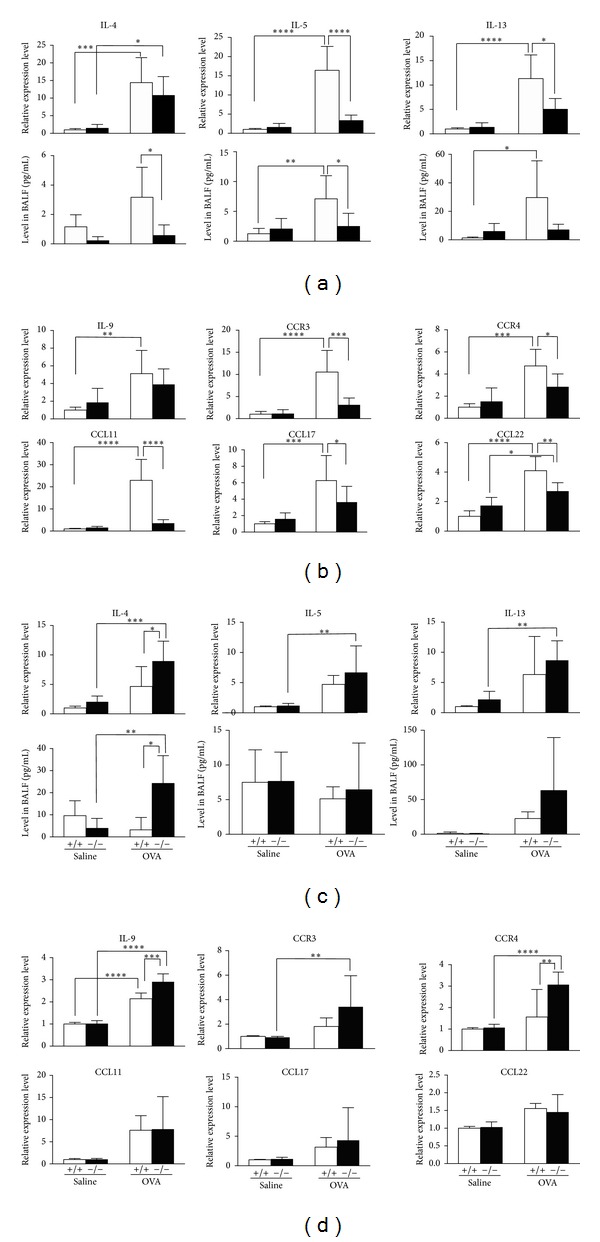
Cytokine expression levels in the lungs and BALF. (a) IL-4, IL-5, and IL-13 expression levels were determined by qRT-PCR for lung mRNA levels (upper panel) and ELISA for protein levels in BALF (lower panel) from control (Saline) and OVA challenged (OVA) *Scgb*3*a*2^−/−(mix)^(−/−) mice and *Scgb*3*a*2^+/+(mix)^ (+/+) mice. (b) Lung mRNAs, encoding IL-9, CCR3, CCR4, CCL11, CCL17, and CCL22 from control (Saline) and OVA-challenged (OVA) *Scgb*3*a*2^−/−(mix)^(−/−) mice and *Scgb*3*a*2^+/+(mix)^ (+/+) mice, were quantified by qRT-PCR. (c) IL-4, IL-5, and IL-13 expression levels were determined by qRT-PCR for lung mRNA levels (upper panel) and ELISA for protein levels in BALF (lower panel) from control (Saline) and OVA challenged (OVA) *Scgb*3*a*2^−/−(N6)^(−/−) mice and *Scgb*3*a*2^+/+(N6)^ (+/+) mice. (d) Lung mRNAs encoding IL-9, CCR3, CCR4, CCL11, CCL17, and CCL22 from control (Saline) and OVA-challenged (OVA) *Scgb*3*a*2^−/−(N6)^(−/−) and *Scgb*3*a*2^+/+(N6)^ (+/+) mice were quantified by qRT-PCR. qRT-PCR results were normalized with* Ppia *and the relative expression levels were expressed based on the value of saline-challenged wild-type lungs as 1. *P < 0.05, **P < 0.01, ***P < 0.001, and ****P < 0.0001 by one-way ANOVA.

The expression of proinflammatory cytokines such as IL-4, IL-5, and IL-13 was determined by qRT-PCR for lung tissue mRNA levels and by ELISA for protein levels in BALF (Figure 4). All three cytokine levels were lower with statistical significance or showed a trend of lower levels in lung tissues as well as BALF from OVA-challenged *Scgb*3*a*2^−/−(mix)^ mice than those of *Scgb*3*a*2^+/+(mix)^ littermates (Figure 4(a)). Levels of mRNAs encoding other cytokines known to be involved in allergic airway inflammation such as CCR3, CCR4, CCL11, CCL17, and CCL22 [16] were also determined by qRT-PCR. Lower levels of these mRNAs were found in OVA-challenged *Scgb*3*a*2^−/−(mix)^ mice than in the *Scgb*3*a*2^+/+(mix)^ littermates (Figure 4(b)). In contrast, for six times C57BL/6NCr backcrossed congenic mice, the level of mRNA encoding IL-4 and IL-4 protein was significantly higher in lung tissues and BALF of OVA-challenged *Scgb*3*a*2^−/−(N6)^ mice, respectively, as compared to those of OVA-challenged *Scgb*3*a*2^+/+(N6)^ mice. IL-5 and IL-13 mRNA and protein levels were similar between the two lines (Figure 4(c)). Further, mRNAs encoding IL-9 and CCR4 were significantly higher in OVA-challenged *Scgb*3*a*2^−/−(N6)^ mice than in OVA-challenged *Scgb*3*a*2^+/+(N6)^ mice (Figure 4(d)). Levels of mRNAs encoding other cytokines, CCR3, CCL11, CCL17, and CCL22, were similar between these two lines of OVA-challenged mice. The results demonstrated that *Scgb*3*a*2^−/−(mix)^ mice are less sensitive to OVA-induced airway inflammation as compared to wild-type controls, while *Scgb*3*a*2^−/−(N6)^ mice exhibited an increased sensitivity to OVA-induced airway inflammation.

## 4. Discussion

This study describes generation and characterization of* Scgb3a2* ^−/−^ mice. These mice were healthy and fertile, demonstrating that SCGB3A2 is not required for development and homeostasis of lung. When subjected to OVA airway inflammation model, however, the mixed genetic background knockout mice exhibited less severe allergic inflammation, while six times C57BL/6NCr backcrossed congenic knockout mice showed similar or increased levels of inflammation as compared to their respective wild-type littermates in terms of lung histology, BALF inflammatory cell numbers, and inflammatory cytokine levels in lung and BALF.

Eosinophil accumulation in the airway wall and lumen is a representative characteristic of asthma [16, 17] and the OVA airway inflammation model mice [6]. Eosinophils are recruited by CCL11 (eotaxin) released by epithelial cells via CCR3 expressed on the cell surface of eosinophils [16]. In our model, while the expression of IL-4 that plays a central role in inflammatory response in allergy was significantly higher in the BALF of OVA-challenged *Scgb*3*a*2^−/−(N6)^ mice compared to that from OVA-challenged *Scgb*3*a*2^+/+(N6)^ mice, the inflammatory indexes determined based on the histological features of lungs and inflammatory cell numbers in BAL were not significantly different between the OVA-challenged *Scgb*3*a*2^−/−(N6)^ and *Scgb*3*a*2^+/+(N6)^ mice. The similar inflammatory indexes found between the two lines of mice may be explained by the levels of CCL11 and CCR3 that were not significantly different between *Scgb*3*a*2^−/−(N6)^ and *Scgb*3*a*2^+/+(N6)^ mice; these two groups of mice had similar levels of eosinophil recruitment.

Whitehead et al. [18] examined the development of airway hyperreactivity and inflammation following OVA sensitization and challenge at various time points (24, 48, and 72 hours after challenge) using nine genetically diverse mice (129/SvIm, A/J, BALB/cJ, BTBR+(T)/tf/tf, CAST/Ei, C3H/HeJ, C57BL/6J, DBA/2J, and FVB/NJ). Following OVA challenge, 129/SvIm and C57BL/6J strains had the greatest number of leukocyte infiltrates in BALF at 72 hours after challenge (~420,000 and ~310,000 cells/mL, resp.), while cells in the other strains were between ~1,000 and ~10,000 cells/mL. As for IL-4 expression, only six of the nine strains sensitized to OVA (129/SvIm, A/J, BTBR+(T)/tf/tf, BALB/cJ, C57BL/6J, and DBA/2J) had significant increases of IL-4 expression in BALF at one or more time points during the postexposure evaluation of 72 hours; the level measured at 24 and 72 hours postexposure was ~35 and 6 pg/mL for 129/SvI and 6 and 4 pg/mL for C57BL/6J, respectively. The results demonstrated a vast difference of inflammatory response, which depended on mouse strains and time points examined. The influence of genetic background on knockout mouse phenotypes has been well documented [19, 20].

*Sc* *gb*3*a*2^−/−(mix)^ and *Scgb*3*a*2^+/+(mix)^ mice used in the current study have mixed background of 129Sv, C57BL/6, and FVB (derived from EIIA-*Cre* mice), each at different percentages between two lines. It is quite possible that, in the OVA-induced allergic airway inflammation model using these mixed background mice, the strain differences may have been a stronger driver than the loss of* Scgb3a2* gene in the development of inflammation, which appeared as if SCGB3A2 played a role in OVA-induced allergic airway inflammation. However, after 6 times backcrossing to C57BL/6NCr, congenic *Scgb*3*a*2^−/−(N6)^ mice showed inflammatory phenotypes that are opposite to those obtained with *Scgb*3*a*2^−/−(mix)^ mice, suggesting that a modifier gene(s) derived from other host genes compared to* Scgb3a2* plays a role in the OVA-induced allergic airway inflammation in *Scgb*3*a*2^−/−(mix)^ mice. Backcrossing of* Scgb3a2* ^−/−^ allele to another strain of mice such as 129Sv and FVB and comparing the phenotypes among the various mouse strains after OVA challenge in combination with genetic mapping may identify potential modifier gene(s) in host mice that influence OVA-induced airway inflammation. Such modifier gene identification was described for polycystic kidney disease using* kat* ^*2J*^ (kat: kidney, anemia, and testes phenotype) mutant mice [21] and multiple intestinal neoplasia using* min *(multiple intestinal neoplasia) mutant mice [22]. On the other hand, a mutation transferred into a different genetic background of mice displays a narrow window of phenotypes, and, depending on the inbred background, this narrow window can be moved toward milder or more severe forms of disease. With a series of different genetic background mice, this may constitute a spectrum of phenotypes in a wide range, which may resemble the symptoms observed for human patients due to their genetic heterogeneity. The identification of modifier genes in mice might provide candidate gene(s) that control the severity of the diseases in humans [19, 20].

Even though the severity of OVA-induced airway inflammation appears to be attenuated in *Scgb*3*a*2^−/−(mix)^ mice as compared with *Scgb*3*a*2^+/+(mix)^ mice, the current results with congenic *Scgb*3*a*2^−/−(N6)^ mice nevertheless suggest that SCGB3A2 is most likely to have an anti-inflammatory activity. This is in good agreement with our earlier study demonstrating that SCGB3A2 exhibits anti-inflammatory activity when OVA airway inflammation model mice were intranasally administered recombinant adenovirus expressing SCGB3A2 [6]. Note that the OVA-induced airway inflammation exhibited by *Scgb*3*a*2^−/−(N6)^ mice was not robust. The reasons for this may be due to the fact that (1) OVA may not be best suited to produce clear differences in experimental model for allergic airway inflammation; (2) in* Scgb3a2* ^−/−^ mice, genes involved in inflammation may have been altered, some of which have compensatory activity to SCGB3A2; and (3) the previous (high level of SCGB3A2 expression obtained by exogenously administered virus) and current experiments (lack of endogenous expression of SCGB3A2) are two different systems, which cannot be directly compared. Other means to produce airway inflammation may be necessary to clearly demonstrate that SCGB3A2 has an anti-inflammatory activity, and lack of this protein makes lung more susceptible to inflammation.

SCGB3A2 was originally identified as a downstream target for the homeodomain transcription factor NKX2-1, one of the critical transcription factors responsible for the development of lung and lung-specific expression of genes [3, 5, 23–25].* Nkx2-1*-null mice had severely hypoplastic lungs with rudimental bronchi [23]. Previously SCGB3A2 was demonstrated to have growth factor activity and to promote embryonic lung development, and administration of SCGB3A2 to* Nkx2-1*-null mice partially restored lung epithelial histology [4]. The reason why naïve* Scgb3a2* ^−/−^ mice did not have any overt histological abnormalities in their lungs is not clear. It may be because SCGB3A2 is among many NKX2-1 downstream targets responsible for the development and homeostasis of lung, which may yield redundancy and overlapping activities, where other gene products could compensate for the deficiency of growth factor activity of SCGB3A2.

## 5. Conclusion

*Scgb3a2* ^−/−^ mice were established and characterized. When subjected to OVA-induced airway inflammation model, *Scgb*3*a*2^−/−(mix)^ and *Scgb*3*a*2^−/−(N6)^ mice showed reduced and increased airway inflammation, respectively. The results suggest that the loss of SCGB3A2 function was influenced by a modifier gene(s) in mixed genetic background and that SCGB3A2 has anti-inflammatory activity. The results demonstrate the importance for the use of congenic knockout mice to understand the role of gene of interest. The results further suggest the possible use of recombinant human SCGB3A2 as an anti-inflammatory agent.

## Figures and Tables

**Table 1:** Primer sets for qRT-PCR.

Gene		Primer set (5′ to 3′)

Il4	Sense	T GAACGAGGTCACAGGAGAA
	Antisense	CGAGCTCACTCTCTGTGGTG

Il5	Sense	GCAATGAGACGATGAGGCTT
	Antisense	CCCACGGACAGTTTGATTCT

Il13	Sense	TGTGTCTCTCCCTCTGACCC
	Antisense	CACACTCCATACCATGCTGC

Il9	Sense	AAGGATGATCCACCGTCAAA
	Antisense	AACAGTCCCTCCCTGTAGCA

Ccr3	Sense	CAGCATGGACGATAGCCAGG
	Antisense	TCAACTTGGCAATTTCTGACCT

Ccr4	Sense	CGACGGCATTGCTTCATAG
	Antisense	GGGTACCAGCAGGAGAAGC

Ccl11	Sense	TCCACAGCGCTTCTATTCCT
	Antisense	TAAAGCAGCAGGAAGTTGGG

Ccl17	Sense	TGCTTCTGGGGACTTTTCTG
	Antisense	ATAGGAATGGCCCCTTTGAA

Ccl22	Sense	TCTGGACCTCAAAATCCTGC
	Antisense	TGGAGTAGCTTCTTCACCCA

Ppia	Sense	GTGTTCTTCGACATCACGGC
	Antisense	CAGTGCTCAGAGCTCGAAAGT
